# RETRACTION: Microglia Play a Major Role in Direct Viral‐Induced Demyelination

**DOI:** 10.1155/jimr/9789048

**Published:** 2026-05-20

**Authors:** Journal of Immunology Research

RETRACTION: D. Chatterjee, K. Biswas, S. Nag, S.G. Ramachandra, and J. Das Sarma, “Microglia Play a Major Role in DirectViral‐Induced Demyelination,” *Journal of Immunology Research*, vol. 2013 (2013). https://doi.org/10.1155/2013/510396.

The above article, published online on 20 June 2013 in Wiley Online Library (wileyonlinelibrary.com), has been retracted by agreement between the Journal’s Editor‐in‐Chief, Nadja Bakocevic; and John Wiley & Sons Ltd.

The retraction has been agreed following an investigation undertaken by the publisher, which identified concerns related to Figure 4. More specifically, the fluorescence microscopy image of microglia shown in Figure 4a is unexpectedly similar with the image of cells shown in Figure 5c from a previous publication by an author group sharing one of the same authors, Jayasri Das Sharma [1].

The authors were contacted and asked to explain this overlap. They claimed that the two images represented the same cell culture and conditions. However, the experiments described in the papers had been conducted at different universities and different growth media were used to culture the microglia. When asked to elaborate on these discrepancies, the authors did not provide a response.

As a result of the investigation, the data and conclusions of this article are considered unreliable. Therefore, the article must be retracted.

The authors disagree with this retraction.
